# The Principles and Practice of Obstetricy

**Published:** 1834-07-01

**Authors:** 


*The Principles and Practice of Obstetricy, as at present taught by* JAMES BLUNDELL, M.D., Professor of Obstetricy at Guy's Hospital. *To which are added, Notes and Illustrations, by* THOMAS CASTLE, M.D., F.L.S.—London, 1834. 8vo. pp. 838.

AMONG those practical problems which are daily debated, yet never solved, there are few more difficult or more important than those which relate to the best method of acquiring the arts and sciences. It is often mooted, for instance, whether the young student should be taught to swim with bladders or without them; whether some kind instructor should ever be at hand to clear up even the shadow of a difficulty, or whether he should be compelled, as soon as possible, to rely on his own unassisted endeavours, and have success, as it were, forced upon him by the lessons which nothing can give like repeated failures. Again, it is often inquired, in our art, whether, after the inculcation of a few theoretical principles, the future physician should be told to rely on the study of nature alone, or whether he is to be perpetually referred for directions to those volumes in which the lights of medicine have recorded their experience. The greater or less value of lectures, too, may very fairly come under discussion: it is often urged against them, that the professor, after all, is merely reading a MS. book to us, and that Denman or Merriman will give us, in a less fleeting form, and for a smaller price, all that can be obtained from the first obstetrical lecturers of the day. To this it may be answered, that many, or most, branches of medicine require the auxiliary aid of drawings and preparations; that it would be infinitely difficult to drive pupils through Smellie or Denman, but that it is comparatively easy to procure their attendance at lectures by statutes for that case made and provided; and, lastly, that a really good lecturer can not only invest the darkest and dreariest parts of our art with the rich hues borrowed from learning and imagination, but can even perform the still more arduous task of adding fresh beauties to those brilliant points which would seem to defy the aid of ornament, and which often form the most disappointing portion of a course, from the exaggerated expectations which they are apt to excite. But these advantages depend on his manner, his animation, his fluency: let his words be printed, and the charm is gone; his minuteness will seem dull, his vivacity may be called petulance; we have the contortions of the Sybil without her inspiration. Discourses must be exquisite, indeed, if they can please when thus dissevered from their better half. A cast from the features of deceased beauty may attract, but who would tolerate the lifeless copy of every-day mediocrity? Hence we are surprised at the custom, which has prevailed for some years, of constantly publishing lectures in the Journals, as if they were the most essential and most agreeable part of the medical literature of the day; and therefore, although we shall notice the work before us, from our respect for Dr. Blundell's reputation as a teacher of midwifery, yet, as the book is professedly elementary, our notice will be a short one, from our respect for our readers.

Dr. Blundell gives the following account of the chief differences between the male and female pelvis.

"The differences which characterize the male or female pelvis are six, more or less considerable.

"First:—In the male there is a certain roughness and bulkiness and weight, which strikingly contrast it with the lighter and smoother and more elegant pelvis of the female. Secondly:—In the male pelvis, the ilia, or wings of the ossa innominata, are more erect; in the female more expanded. Thirdly:—in the male the brim is more rounded, though tending somewhat to an ellipse, the long diameter of which stretches from before backward; in the female, the brim, though sometimes rounded, is generally oval, and the long diameter lies between the sides. Fourthly:—Male pelvis deep; female pelvis shallow. Fifthly:—The male outlet very small; the female outlet very capacious. Sixthly:—In the male, the arch of the pubes is contracted; in the female it is capacious, to make room for the more ready passage of the head." (P. 20.)

Soemmerring, who, in his account of the measurements of the pelvis, has copied a previous writer, tells us that the great diameter of the brim of the pelvis, or, in other words, the distance between the ossa iliûm, should, in the female, be five inches and six lines; and the smaller diameter, or distance from the sacrum to the os pubis, should be four inches and eight lines: while the former diameter, in the male, is four inches and six lines, and the latter from four to five inches. (*S. Th. Soemmerring, de Corporis Humani Fabricâ,* tom. i. § 446.) It would seem doubtful, therefore, whether, in the opinion of this great anatomist, the diameter from ilium to ilium, or from sacrum to pubes, be the greater in the male.

We do not know what inches Soemmerring (or the author whom he transcribes,) employed in his measurements, which will consequently show only the relative, and not the absolute, diameters of the pelvis.

It is not surprising that the duration of pregnancy should still be a matter of dispute, as opportunities of ascertaining it with scrupulous exactness are few and far between; and writers, gratified with the cases in which it was possible to do so, have too hastily built a theory on two or three examples, and have probably sometimes elevated the exception into the rule. Dr. Blundell thinks that the most usual duration of pregnancy is thirty-nine weeks, plus one day; he says , "I was surprised to learn that, in a late investigation before the tribunal of the empire, nine months of the calendar, and forty weeks, were, by some of the witnesses, used interchangeably, as if they were commensurate periods: the error will appear on a little calculation, as the period of nine months is exactly equal to that of thirty-nine weeks, plus one day, provided of these nine months five are of thirty days only, and four of thirty-one." (P. 175.) These witnesses would certainly not have been qualified to revise a new edition of Scaliger de Emendatione Temporum; but our author has fallen into an error of the same class, as it is impossible to pick out nine consecutive months, of which five shall have thirty days, and four thirty-one. Our opinion is, that the duration of pregnancy may vary considerably; a supposition backed not only by the unfixed duration in animals, but by the analogous irregularity of the appearance and disappearance of the catamenia in women.

When will some accoucheur arise of sufficient courage to avow, that in natural labour there is nothing to be done, and that the duty assigned him of supporting the perineum is an imaginary one? And when shall we have a race of accoucheurs who will instruct their patients, that perpetual examinations are worse than useless, and that, in ordinary cases, tincture of time (as Gooch facetiously called it,) is the only remedy required? More than half our progress in knowledge consists in unlearning deeply-rooted errors: the poor wear the cast-off opinions of their betters, as well as their clothes, and the piercing cries of "Pray help me, doctor!" are in part the distant echoes of worm-eaten treatises on midwifery.

We do not think that our author will be offended with these strictures, as he has given us a beautiful panegyric on truth, couched in a loftier style than we are accustomed to see in medical writings.

"From the most violent conflicts of opinion, truth has nothing to fear; though long to us, to her a thousand years are but as one day, a point, a nothing in the eternity of her duration. Oppressed, amongst us, beneath the chaos of human follies and errors, she must, she will emerge unhurt at last, unchangeable as her Author. By the mere force of durability, she must ultimately stand alone, solitary amid the wreck of those perishable materials, by which, for a time, she is overwhelmed—'and the ark floated in the midst of the waters.' To her, the living spirit of philosophy, immutable, immortal, infinite, eternal truth, to her, parent of all knowledge, fountain of light, to her may be addressed, without perversion or hyperbole, the sublime apostrophe of the poet,

'The stars shall fade away, the sun himself
Grow dim with age, and nature sink in years,
But thou shalt flourish in immortal youth,
Unhurt.'" (P. 428.)

The operation (as it is called) of Embryotomy forms the subject of perhaps the most difficult question in the whole range of medical ethics. Is it allowable to sacrifice one life to preserve another? or, on the other hand, shall we suffer a woman to perish, when she might be saved by the extinction of the half-existence of a being yet unborn? Our readers well know that the answers to these questions depend entirely on the countries in which they are asked; and that British accoucheurs stand almost or quite alone in their readiness to perform embryotomy. We side with them in those cases where the pelvis will certainly not afford a passage to the child; but we think that the operation cannot be justified by the mere apprehension that the mother *may* sink through the protraction of the labour, or *may* be disfigured by the laceration of the parts. We therefore do not quite agree with our author when he says, "that, in British obstetricy, the life, nay, the preservation of the patient from the graver lesions of her person, is to be looked upon as paramount to every consideration relating to the fœtus; and, when these require the sacrifice, craniotomy becomes justifiable." (P. 546.)

In the section on the means of superseding the Cæsarian operation, Dr. Blundell makes a bold proposal, which will probably be carried into effect some day in these operation-seeking times, to use a phrase of Dr. Jörg, *(in diesen operationssüchtigen Zeiten.)*

"Now, is there any mode in which, when the obstruction of the pelvis is insuperable, the formation of a fœtus may be prevented? In my opinion there is: for if a woman were in that condition, in which delivery could not take place by the natural passage, provided she distrusted the circumstances in which she was placed, I would advise an incision of an inch in length in the linea alba above the symphysis pubis; I would advise, further, that the fallopian tube on either side should be drawn up to this aperture; and, lastly, I would advise, that a portion of the tube should be removed, an operation easily performed, when the woman would, for ever after, be sterile." (P. 580.)

Dr. Blundell is well known to be a zealous advocate of transfusion, and in the present lectures he has given the theory and practice of this ingenious but dangerous operation at some length. After showing that the blood of one animal may be substituted for the blood of another of the same species, while that of animals of a different species is destructive; and that blood may be transmitted through a syringe without much deterioration, our author gives the following account of the mode of performing transfusion.

"There are different ways in which transfusion may be performed; and I shall first briefly state to you the method approved now by experience, and which, perhaps, for general purposes, may at present be deemed the best. And first, then, the operation may be executed by means of a well-constructed two ounce syringe, air secure, made of brass, tinned internally, not offensive with oil, of course perfectly clean, and to be used in the following manner: One or two bystanders (males flow more copiously than females) being in readiness to supply the requisite quantity of blood, the arm of the patient should be prepared as follows: taking a scalpel, at one cut, if tolerably dexterous, you lay bare the bleeding vein, which opens on the eye under the knife, the patient being so far from suffering in this part of the operation, that frequently she is not aware that it has been done. The vessel manifesting itself, you take a short curved probe, which you slide beneath it at the lower extremity of the incision, with a well-sharpened lancet, laying open the vein to the extent of about a line, that is, one-eighth of an inch; afterwards introducing, cautiously, at this orifice, the tubule of the syringe, so as to satisfy yourselves that when you operate the entrance will be easy; at this time, perhaps, a little blood oozes out. This preparation made, you bind up the arm of the person who is to yield the supply of blood, laying open the vein in the usual manner, but making the orifice rather free. In a conical tumbler, of large diameter, the blood may be conveniently gathered; and into the syringe, previously washed and warmed by transmission of water milk-warm, the blood is to be absorbed from the point of the tumbler through the long tubule, in such manner that, although the whole of the blood is not to be taken up lest the air should be drawn in, not more than a dessert spoonful is to be allowed to accumulate at once in the bottom of the vessel; in truth, it is not in the glass, but in the barrel of the syringe that the blood should collect. This tubule should be long enough to throw the barrel of the syringe above and beyond the brim of the tumbler, so that it may be completely out of the way. That it may enter the vein more easily, the end of the tubule may be bevelled, like the tea-pot spout.

"Two ounces of blood from the arm being absorbed in this manner, holding the syringe vertically with the tubule above and the handle of the piston below, you slowly urge the piston onward, till, together with all air, about a dessert spoonful of blood has been expelled; and then closing the nozzle by the apposition of the tip of the finger, lest the piston descending by its own gravity, fresh air should be absorbed, you give the instrument the horizontal direction, and proceed to insinuate the blood into the vein. On approaching the arm of the patient perhaps you find the orifice obscured by the blood; touch the vein with a sponge, and the aperture may be seen as clearly as the letter of a book. At this time an assistant may gently press the vein, where it lies across the probe, which will intercept a further exudation, for the circulation is so low that it is easily arrested. These preliminaries premised, without trepidation, with that calm and measured movement of mind and body, the result, not of mere animal spirits, but of that confidence which arises from a mind well prepared, you proceed to deliver the blood, cautious not to interpose unnecessary delay. For this purpose, the tubule being insinuated into the vein, to the extent of half an inch towards the heart, it is your next office to infuse the blood into the vessel, and very nice and critical is this point of the operation. What the heart in women or men might bear in a state of vigour I do not know, but reduced as it is in these cases, feeble as the limb which refuses to sustain them, it cannot support a sudden influx of the blood. To infuse too slowly is an error no doubt, for, while lying in the syringe, the blood every moment is becoming more and more deteriorated; but to inject too rapidly is a still more fatal error: gorge the cardiac cavities, and the patient may perish as suddenly as if shot through the heart; it is, therefore, with moderate velocity that the blood should be infused, and most cautiously when the collapse is great. In pressing forward the piston, from moment to moment, fix your eye on the countenance, and if all is well, then proceed more boldly; but if the lip quiver, or the eyelid flicker, or if there be restlessness or vomiting, though these are not fatal symptoms, yet it is better to suspend your operation until they subside, as in the present state of our information there is good cause for alarm; and let me add, that after waiting in this manner, you must not return to the injection, until you have procured a fresh supply of blood. If the first two ounces load, it is better to wait a few minutes, say six or eight, before more is injected; but if these first two ounces are well received by the system, proceed immediately to inject other two afterwards, waiting for eight or ten minutes, till the whole have duly circulated over the body, and, in some measure, at least, have renewed its vigour; under the extremes of weakness, this caution becomes especially necessary. Sixteen ounces of blood for the female system is a large measure, eight or ten are more sparing; four or five may, in delicate cases, turn the scale in our favour. If our object is simply to save life, the smaller quantities must be injected; if to restore vigour, the larger. Whether we transfuse or not after floodings, re-action is apt to come on next day. The entrance of a single bubble of air, though not fatal, is always to be deprecated. Inflammation of the vein is a neat topic of declamation; after the danger is blown over, listen with decent attention; till then you have not time to think about it, Antipater, and the myo-machia, may cross the classical mind. If the blood dribble from the arm which supplies you, or if it be slightly coagulated, it is unsafe, if not wholly unfit, for use. Wash the syringe between each injection. Watch the arm lest it inflame afterwards. If the respiration be stopped, it is, I fear, in vain to transfuse; if respiration is at its last gasp, the hope is small, a sudden influx of two ounces would, I think, certainly destroy in these cases. Would the heart bear, at proper intervals, doses of half an ounce? If the respiration be steady, you are almost certain of success. The best syringes I know of are those of Laundy, Weiss, Reid, and Scott. Laundy's are made according to my own whim; of course I think them preferable. Transfusion from artery to vein, perhaps even from vein to vein, might be accomplished by tubule simply; could you, however, obtain readily those who would supply you in this mode, the arterial transfusion especially would require caution; if the heart were very feeble, an impetuous influx would destroy." (P. 423.)

Dr. Blundell has given a long account of what he calls Hidrotic Fever: it is a low fever occurring after parturition, and accompanied with sweats, (whence its name, from ἱδρως or ἱδρωτικος.) Our author has divided this disease into seven kinds; yet, in spite of the pains he has taken with it, we venture to prophesy, that this new malady will not be able to hold up its head among its fellow-fevers. It seems to us to want vivaciousness.

These lectures are upon the whole very good ones, and, though they cannot aspire to supersede the established text-books, will yet deserve a place in an obstetrical library. Dr. Castle has performed his task with diligence and fidelity: his notes will be useful to beginners, and show very laudable industry. Considering, however, that his preface is dated from Trinity College, Cambridge, we could have wished his Greek and Latin to have appeared in a more polished state. He might have found at least two hundred undergraduates in that learned body who would have told him that the latter half of *perinæum* is not derived from νεω, to accumulate, as he supposes at p. 32 *(note)*, but from νεω, to flow; and that *ovarium* is not a *diminutive* of *ovum*, as he asserts at p. 37 *(note)*, but signifies an egg-bed, just as *rosarium* means a rose-bed. We hope that he will take these friendly hints in good part, and recollect that we expect men to return with gold from Peru, and with knowledge from Cambridge.

